# Transformative weight loss with Dulaglutide: A case report of success in a challenging patient profile of an ex‐sumo wrestler

**DOI:** 10.1002/ccr3.9403

**Published:** 2024-08-29

**Authors:** Sohail Bade, Sahil Bade, Grishma Sharma, Narayan Bhurtel, Yadvinder Singh, Sudip Paudel, Frena Pulami Magar, Kshitij Chapagain

**Affiliations:** ^1^ Dhulikhel Hospital Dhulikhel Nepal; ^2^ Kathmandu Metro Hospital Kathmandu Nepal; ^3^ Swacon International Hospital Kathmandu Nepal; ^4^ Tribhuvan University Teaching Hospital Kathmandu Nepal; ^5^ Singapore Gurkha Polyclinic Ltd Lalitpur Nepal

**Keywords:** dulaglutide, GLP‐1 receptor agonist, obesity, type 2 diabetes mellitus, weight loss

## Abstract

**Key Clinical Message:**

Dulaglutide is a relatively unpopular GLP‐1 receptor agonist used for weight loss. This case demonstrates that dulaglutide may be beneficial for weight loss in morbidly obese patients with multiple comorbidities after thoroughly evaluating its efficacy, benefits, and long‐term adverse effects through clinical trials.

**Abstract:**

We present a case of a 27‐year‐old ex‐sumo wrestler with bipolar II disorder, morbid obesity, hypertension, Type 2 Diabetes Mellitus (DM), and a Body Mass Index (BMI) of 49.66 kg/m^2^. He was non‐compliant with lifestyle modifications and resistant to conventional treatments, including metformin, and was also using multiple antipsychotic drugs. After introducing dulaglutide, he achieved a 40 kg (−21%) weight loss and a BMI reduction of 10.3 kg/m^2^ over 6 months, with no side effects and improved glycemic control, demonstrating dulaglutide's efficacy for weight loss in such challenging presentations.

## 1 INTRODUCTION

The global prevalence of overweight and obesity (Body Mass Index (BMI) ≥25 kg/m^2^) is expected to increase from 38% to 50% between 2020 and 2035 with a rise in the number of affected individuals from 2.6 billion to 4 billion.^1^ Overweight and obesity is a rising public health problem due to its associated increased risks of chronic health problems including coronary heart disease, type 2 diabetes, stroke, hypertension, sleep apnea, dyslipidemia, and some cancers.^2^

In patients with Type 2 Diabetes Mellitus (Type 2 DM), even modest amounts of weight loss as low as 5% have shown to be beneficial for glycemic control, control of blood pressure, and lipid level management, thereby reducing cardiovascular risk. A prospective 12‐year follow‐up study in 4970 overweight individuals with diabetes showed that weight loss was associated with a 25% reduction in total mortality, highlighting its importance in diabetic management.^3^

Although lifestyle interventions like dietary modifications, increasing physical activities, and bariatric surgery are classical approaches for weight reduction, the cumbersome requirement of commitment to lifestyle modification and associated negative postoperative outcomes has caused a growing interest in pharmacological approaches to weight loss.^4^ GLP‐1 receptor agonists (GLP‐1 RA), an analog of human glucagon‐like peptide‐1, is an incretin mimetic hormone that acts by increasing insulin secretion, decreasing glucagon secretion, inhibiting postprandial gastric emptying which decreases appetite and lowers body weight.^5^, ^6^

Dulaglutide is a GLP‐1 RA that has been shown to exert great potential for inducing weight loss in patients with Type 2 DM, particularly in cases where conventional anti‐obesity therapies have proven insufficient.^7^, ^8^, ^9^ However, its applicability for weight management is poorly explored for challenging patient profiles such as those with multiple comorbidities. We present a 27‐year‐old ex‐sumo wrestler with a history of bipolar II affective disorder, morbid obesity, hypertension, and Type 2 DM, who achieved substantial weight loss of 40 kg over 6 months after initiation of dulaglutide injection. This report is significant and novel as it demonstrates the potential of dulaglutide for weight management in patients with multifaceted challenges of real‐world scenarios such as multiple comorbidities, non‐compliance to conventional treatments, and combating weight gain associated with concomitant use of psychiatric medications. Additionally, the 40 kg weight loss in our ex‐sumo wrestler with dulaglutide is unprecedented and not previously documented.

## 2 CASE REPORT

### 2.1 Clinical history

A 27‐year‐old male with a history of bipolar II disorder, hypertension, Type 2 DM, and alcohol use disorder presented to the emergency room with tremors in his hands and suicidal thoughts lasting 3 days, accompanied by attempts of self‐harm. He appeared restless and irritable but denied symptoms such as palpitations, sweating, altered bowel habits, heat/cold intolerance, headache, chest pain, or changes in sleep patterns. He admitted to excessive alcohol consumption over the past week due to relationship issues.

His alcohol use began at age 14, escalating significantly during his two‐year stay in Japan at age 20 training as a sumo wrestler. Following intentional surplus calorie intake, his weight increased from 132 kg to 185 kg in that period, and he experienced mood swings and sleep disturbances, leading to a diagnosis of bipolar II disorder and alcohol use disorder. At age 22, he was also diagnosed with type 2 diabetes and hypertension.

He has been taking sodium valproate 1 g twice daily, metformin 500 mg twice daily, lamotrigine 200 mg once daily, aripiprazole 30 mg once daily, losartan 50 mg once daily, and clonazepam 0.5 mg at bedtime for the past 5 years. Despite attempting lifestyle and dietary changes to the Mediterranean diet for the past 5 years, the patient mentioned struggling to lose weight as he failed to adhere to and maintain them, particularly during periods of psychiatric episodes.

### 2.2 Examination

His vital signs were within normal limits. A physical examination revealed mild dehydration and multiple cut injuries over the ventral aspect of the bilateral forearm in a healing state. His central nervous system was grossly intact with a GCS score of 15/15. His other systemic examination revealed no abnormalities. His anthropometric measurements were a weight of 185 kg, a height of 193 cm, and a BMI of 49.66 kg/m^2^.

### 2.3 Investigations

His lab workup revealed a total leucocyte count of 9800/mm^3^ with eosinophilia (12%), low platelet (1,29,000/mm^3^), a random blood sugar of 128 mg/dL, sodium of 143 mEq/L, potassium of 4.2 mEq/L, urea 28 mg/dL, creatinine 0.51 mg/dL, LDL 125 mg/dL, HDL 48 mg/dL, Total Cholesterol 180 mg/dL, Triglyceride 145 mg/dL, HBA1c‐ 5.5% with normal liver function test, thyroid function test, urine routine and echocardiography findings. His serology for HCV, HIV, and HBs antigens was negative. Ultrasonography of the abdomen and pelvis revealed borderline hepatomegaly with moderate to gross fatty infiltration, and bilateral large kidneys with normal corticomedullary differentiation.

### 2.4 Diagnosis and management

He was admitted to the general ward, a psychiatry consultation was done, and diagnosed with Alcohol Dependence Syndrome with Bipolar II affective disorder. He was treated with intravenous normal saline, alprazolam, disulfiram, olanzapine, pantoprazole, buscopan, and amlodipine. His regular medications like valproic acid, metformin, lamotrigine, aripiprazole, clozapine, and losartan were continued during the stay. He improved symptomatically throughout the hospital stay.

The patient had faced significant challenges in adhering to a Mediterranean diet and maintaining a regular exercise routine due to recurrent episodes of mood disorder. These challenges were exacerbated by weight gain associated with the use of olanzapine and sodium valproate for 5 years. Despite the introduction of metformin, the weight gain issue persisted, indicating the need for an additional therapeutic approach. Therefore, we decided to counsel the patient on weight reduction through the administration of the GLP‐1 agonist dulaglutide. The off‐label use of this medication and its potential side effects were thoroughly explained to the patient and their caregiver.

Following consent, the patient commenced treatment with an Injection of dulaglutide 0.75 mg subcutaneously once weekly, with a subsequent dose escalation to 3 mg once weekly over a span of 6 weeks. Following treatment initiation, the patient reported increased satiety and reduced calorie intake. Dulaglutide was administered for a total of 6 months, during which compliance was ensured with proper documentation of regular visits and drug administration. The patient underwent close monitoring for any potential side effects. Patient calorie intake along the course of the treatment plan is mentioned below. (Table 1).

**TABLE 1:** Patient calorie intake before and during treatment.

	Morning	Afternoon	Night	Daily total calories
Before treatment	1512	3900	1512	6924
2 weeks after treatment	978.9	2925	844.92	4748.8
2 months after treatment	810	1480	810	3100
6 months after treatment	781.95	975	781.95	2538.9

Assessments including a lipid profile, HbA1C, and abdominal ultrasound were conducted before the administration of dulaglutide and 6 months after, revealing no signs of adverse effects or complications or any significant changes. The patient did not experience any episodes of adverse reactions such as diarrhea, vomiting, abdominal pain, hypoglycemia, or any episodes of pancreatitis or acute kidney injury throughout the treatment period. The doses of other medications remained constant throughout the 6 months.

**FIGURE 1:**
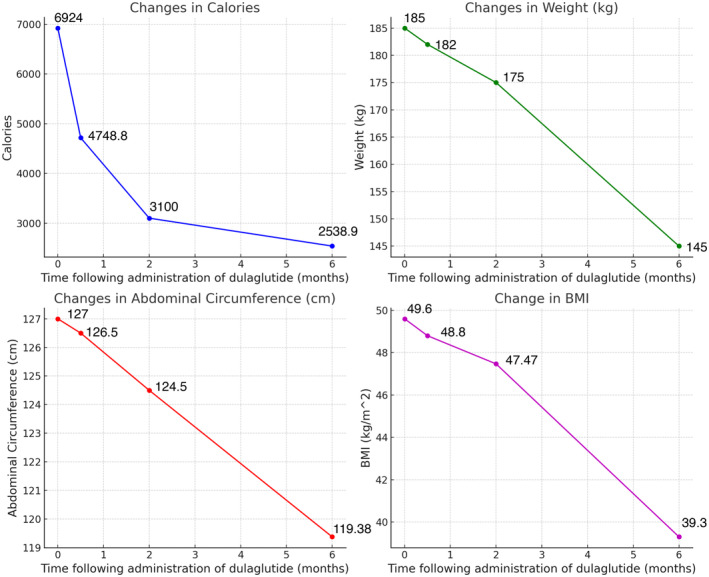
Graphs showing changes in calorie intake, weight, BMI, and abdominal circumference over 6 months after dulaglutide administration.

After 6 months of treatment with dulaglutide, the weight of the patient decreased from 185 kg to 145 kg with 40 kg (−21%) weight loss and a decrease in BMI by 10.3 kg/m^2^ and abdominal circumference by 7.62 cm. Throughout this period his daily calorie intake significantly decreased from 6924 to 2538. This reduction was primarily due to a notable decrease in appetite throughout the day and an increased feeling of satiety even after consuming a smaller amount of food. The subjective quantification of hunger and satiety was done using a visual analog scale. Assessments were conducted weekly, first in a fasting state every morning at 7 a.m., and again in a postprandial state starting immediately after finishing a meal, with evaluations every 20 min for the next 2 h. A fixed overnight fasting time and the intake of comparable quantities of rice, pulses, and vegetables at dinner and lunch on every assessment day were ensured through video‐assisted supervision. (Table 2) (Figure 1).

**TABLE 2:** Changes in weight, height, BMI, and abdominal circumference over 6 months of treatment.

	Weight (kg)	Height (cm)	BMI (Kg/m^2^)	Abdominal circumference (cm)
Before treatment	185	193	49.6	127
After 2 weeks of treatment	182	193	48.8	126.5
After 2 months of treatment	175	193	47.47	124.5
After 6 months of treatment	145	193	39.3	119.38

## 3 DISCUSSION

In this case report, we present the management of a 27‐year‐old male diagnosed with bipolar affective disorder type 2, hypertension, Type 2 DM, morbid obesity, and alcohol use disorder, who presented with tremors and suicidal thoughts. With a history of intentional weight gain during his pursuit of becoming a sumo wrestler in Japan, the patient faced challenges in weight management despite lifestyle modifications and pharmacotherapy. Given his lack of compliance with conventional weight loss interventions, the GLP‐1 RA dulaglutide was introduced as an adjunctive therapy as an add‐on to Metformin. Over 6 months, the patient exhibited favorable responses to dulaglutide, reporting increased satiety and reduced calorie intake resulting in weight loss of 40 kg and a decrease in BMI by 10.3 kg/m^2^. The temporal relationship between the start of dulaglutide and subsequent weight loss, marked increase in satiety as evident by visual analog scale consistent with known mechanism of GLP‐1 RA, in a scenario of no new lifestyle modification and constant doses of psychiatric and anti‐diabetic medication suggests that dulaglutide was the primary factor contributing to the significant weight loss in the patient.

The FDA‐approved long‐term weight‐loss medications in the United States include orlistat, lorcaserin, naltrexone‐bupropion, topiramate, phentermine‐topiramate, semaglutide, and liraglutide. The GLP‐1 RA semaglutide and liraglutide, which are approved for the treatment of obesity in the United States, are injected subcutaneously. Because of their proven ability to cause weight loss and their well‐tolerated safety profiles, they are advised as first treatments for obesity. A consensus report by the American Diabetes Association and European Association for the Study of Diabetes recommends GLP‐1 RA as second‐line therapy after metformin in individuals with Type 2 DM when choosing glucose‐lowering medication, particularly if there is a compelling need to minimize weight gain or promote weight loss.^10^

Although having received FDA approval primarily for glycemic control in patients with Type 2 DM, dulaglutide has also garnered attention for its adjunctive role in weight management. Recent studies, such as the AWARD 11 Trial, have demonstrated the efficacy of dulaglutide in promoting weight loss in individuals with obesity.^11^ A meta‐analysis involving 18 studies done in Type 2 DM patients demonstrated a significant decrease in body weight, BMI, and waist circumference following subcutaneous dulaglutide administration compared to placebo.^9^ A study analyzing the effect of dulaglutide at 26 weeks following initiation of the drug in Award 1–6 clinical trial showed dulaglutide 1.5 mg caused significantly greater reduction in weight and HbA1c as compared to metformin, sitagliptin, exenatide twice daily, and insulin glargine. Similarly, 0.5 mg of dulaglutide had a significantly greater reduction in weight and HbA1c as compared to sitagliptin and insulin glargine and a comparable effect with Metformin and Exenatide twice daily.^12^ A systematic review including three trials demonstrated that one weekly dulaglutide is associated with a greater reduction in HBA1c compared to once daily Liraglutide. Dulaglutide also had significantly lower gastrointestinal side effects and caused less increase in heart rate as compared to liraglutide.^8^

Close monitoring of our patient revealed no adverse effects or complications, with no episodes of hypoglycemia observed. However, it's crucial to note the potential side effects of dulaglutide, including gastrointestinal symptoms like nausea, diarrhea, vomiting, abdominal pain, decreased appetite, dyspepsia, fatigue and rare occurrences of pancreatitis, medullary thyroid carcinoma, PR interval prolongation, first‐degree atrioventricular block. The most common side effects of dulaglutide are nausea, vomiting, and diarrhea.^13^ Although greater reduction in body weight was seen in those who developed nausea and/or vomiting, the reduction was seen irrespective of nausea and/or vomiting. The incidence of nausea rapidly declined after the first 4 weeks yet weight change continued well beyond this time indicating multiple mechanisms of dulaglutide in weight loss.^12^

Incorporating dulaglutide into the treatment regimen of diabetic patients struggling with obesity and its associated complications offers a promising avenue for holistic management as weight loss along with glycemic control is one of the important aspects of diabetes management. However, it warrants further exploration and consideration in clinical practice considering limited research on its long‐term efficacy and side effects and its limited role in reducing cardiovascular risk and slowing diabetic nephropathy progression compared to other GLP 1 RA like liraglutide.^14^, ^15^, ^16^ The limitation of our study is that our case of a single patient will not provide sufficient evidence to capture possible short‐term side effects, even though our patient did not develop any complications. Furthermore, the limited follow‐up time of 6 months in our patient makes it challenging to monitor the efficacy and development of possible long‐term side effects related to dulaglutide use.

## 4 CONCLUSION

Dulaglutide, a convenient weekly injectable with strong glycemic efficacy and potential for weight loss, emerges as a promising new GLP‐1 RA for the treatment of obesity even in patients with multiple comorbidities. The 40 kg weight loss in our ex‐sumo wrestler with dulaglutide is unprecedented and not previously documented. Therefore, the clinical implication of this case highlights the potential of dulaglutide as an effective therapy for weight management in patients with multifaceted challenges of real‐world scenarios such as multiple comorbidities, non‐compliance to conventional treatment, and combating weight gain associated with concomitant use of psychiatric medications, though larger‐scale studies are warranted to confirm these observations and guide clinical practice.

## AUTHOR CONTRIBUTIONS

**Sohail Bade:** Conceptualization; data curation; supervision; validation; writing – original draft. **Sahil Bade:** Conceptualization; data curation; supervision; validation; writing – original draft. **Grishma Sharma:** Conceptualization; data curation; supervision; validation; writing – original draft. **Narayan Bhurtel:** Conceptualization; data curation; supervision; validation; writing – original draft. **Yadvinder Singh:** Writing – original draft. **Sudip Paudel:** Writing – original draft. **Frena Pulami Magar:** Writing – original draft. **Kshitij Chapagain:** Writing – original draft.

## FUNDING INFORMATION

None.

## CONFLICT OF INTEREST STATEMENT

None declared.

## ETHICS STATEMENT

Ethical approval was not required for the case report per the country's guidelines.

## CONSENT

Written informed consent was obtained from the patient to publish this report in accordance with the journal's patient consent policy.

## PREPRINT

Preprinted on Authorea‐ Date July 16, 2024. DOI: https://doi.org/10.22541/au.172115447.70859792/v1.

## Data Availability

The data supporting this article's findings are available from the corresponding author upon reasonable request.
